# Laboratory assessment of SD Bioline HIV/Syphilis Duo Kit among pregnant women attending antenatal clinic Mayuge Health Center III, East central Uganda

**DOI:** 10.1186/s13104-019-4272-6

**Published:** 2019-04-25

**Authors:** Ivan Mugisha Taremwa, Alupakusadi Twelwanike, Bashir Mwambi, Christine Atuhairwe

**Affiliations:** 1 Department of Medical Laboratory Sciences, Clarke International University, P.O Box 7782, Kampala, Uganda; 2 Institute of Public Health and Management, Clarke International University, P.O Box 7782, Kampala, Uganda

**Keywords:** HIV, Screening, Syphilis, SD Bioline Duo assay, Uganda

## Abstract

**Objective:**

Efforts to dual eradication of mother-to-child transmission of human immune deficiency virus (HIV) and syphilis have improved in the previous decades. This has however been hindered by limited validation studies. A cross-sectional study was conducted among adult pregnant women attending antenatal care clinic at Mayuge Health Center III. Two milliliters of venous blood were collected into Ethylene di-amine tetra acetic acid vacutainers, and tested for HIV and syphilis using the SD Bioline HIV/Syphilis Duo assay, and the national HIV and syphilis testing algorithm. Sensitivity and specificity were calculated for the Duo Kit against the gold standards within 95% confidence intervals.

**Results:**

Three hundred and eighty-two (382) participants were enrolled. Their mean age was 25.8 years. The prevalence of HIV was 1.8% (95% confidence interval 1.23–2.41); while that of syphilis was 2.1% (95% confidence interval 1.81–2.54), and the dual infection was 0.52% (95% confidence interval 0.37–0.92). The sensitivity and specificity of the SD Bioline HIV/Syphilis Duo assay were all 100.0% (95% confidence interval 99.5 to 100.0 and 98.6 to 100.0, respectively). The performance of the SD Bioline HIV/Syphilis Duo Kit was optimal, reassuring its aptness for use, and favorable qualities to a limited resource setting.

**Electronic supplementary material:**

The online version of this article (10.1186/s13104-019-4272-6) contains supplementary material, which is available to authorized users.

## Introduction

The burden of HIV and syphilis infections remains high, with an estimated rates of 1.5 and 1.36 million pregnant women respectively [1–3]. Africa accounts for 42.4% of the total HIV/Syphilis burden [4], with pregnant women and newborns being amongst the at risk populations [5]. The prevalence of syphilis among pregnant women attending antenatal care in Uganda has been reported at 6.4% [6], while that of HIV at 6% [7]. The maternal effects of untreated syphilis are devastating, with pregnancy adverse outcomes like spontaneous abortion, stillbirth, fetal death, preterm birth, low birth weight and high risk of mother-to-child transmission (MTCT) [8, 9].

While antenatal syphilis and HIV screening is a policy in most countries [9], syphilis testing remains under-sourced, and infected pregnant women often go undiagnosed and untreated [9]. Although multifactorial, routine syphilis screening is limited by funding and logistical drawbacks [5], and methodical single testing approaches that are often expensive [4]. To bridge the low syphilis test coverage and maximize the benefits of early HIV and syphilis testing, the World Health Organization (WHO) has commended HIV and syphilis dual screening in a combined test as part of ANC program [3, 7, 10–12]. In light of this, a point-of-care test, the SD Bioline HIV/Syphilis Duo test (*Standard Diagnostics, Korea*) has been invented [13]. Studies to evaluate the diagnostic performance of SD Bioline HIV/Syphilis Duo Kit among pregnant women have demonstrated a good clinical performance in diagnosing both HIV and syphilis [14–19]. This coupled with other attributes like being user friendly, a shorter turnaround time of 25 min and relative costs approximated to 2 USD per test [14] renders it apt. Although the SD Bioline HIV/Syphilis Duo Kit is WHO prequalified [19], its diagnostic performance has not been widely evaluated. This study determined the diagnostic performance of SD Bioline HIV/Syphilis Duo Kit among pregnant women attending antenatal care clinic at Mayuge Health Center III, Mayuge district, East central Uganda.

## Main text

### Materials and methods

It was across-sectional study conducted at Mayuge Health Center III, antenatal care unit during the months of July to September, 2018. The facility offers varied services, including antenatal care, and has admission capacity of about 12–18 patients daily. It is located along Musita-Lumino Busia highway Mayuge district, Mayuge Town council, Busoga sub-region in East-central Uganda. It supports the neighboring districts of Bugiri, Iganga and Jinja.

The study population comprised consented pregnant women aged 18–49 years, who did not know their HIV and syphilis sero-status. The study excluded pregnant women who reported with a known positive HIV or syphilis test and obstetric emergency. Participants who were HIV and syphilis positive sero-positive were channelled directly to care. Sample size was determined using Kish Leslie formula [20], given as n = Z^2^pq/d^2^. Using 50% estimated proportion of HIV/Syphilis co-infection and an allowable error of 5%; a sample size of 382 was considered.

**Fig. 1:**
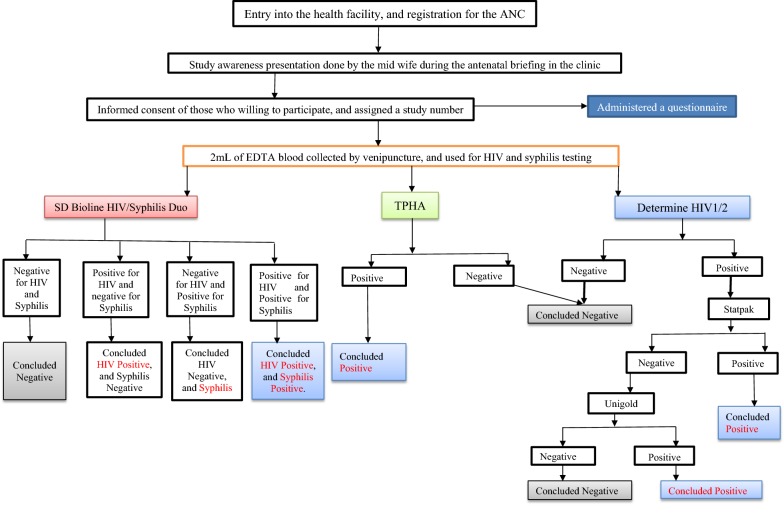
showing participant enrolment and testing flow

The study used an-interviewer administered questionnaire and laboratory analysis of blood samples to obtain data. The questionnaire captured socio-demographic and exposure history of the study participants. In addition, the three laboratory personnel involved in the sample analyses were given feasibility questionnaires to assess the usability of the SD Bioline HIV/Syphilis Duo Kit compared to the routine single HIV and syphilis test methods. This focused on the ease of the test method, mean time to results, sample volumes used in each assay, and ease of result interpretation. The laboratory assays were done by two laboratory technicians attached to the Mayuge Health centre III. For the purpose of this research, these technicians were trained on the use of SD Bioline HIV/Syphilis Duo assay prior to initiation of the study. On a single day, one technician performed all the SD Bioline assays, while the other performed the Determine/Statpak/Unigold for HIV, and TPHA for syphilis. These were rotated daily, and a third technician (Principal Investigator) oversaw the entire process. The two laboratory technicians were blinded to each other’s results, and a third reader (Principal Investigator) was considered for discrepant results of the two test assays, and also to resolve such results that were not obvious to conclude. About 2 mL of blood samples were collected using Ethylene di-amine tetra acetic acid (EDTA) vacutainer. The laboratory technician attached to the antenatal care clinic laboratory used about 20 µL of the venous blood to perform the SD Bioline assay. The remaining blood samples were transported in a cool box maintained at 2–8 °C using frozen icepacks to the HIV clinic where they were separated by centrifugation (Thermo SCIENTIFIC, CL10 Centrifuge) at 3000 revolutions per minute for 10 min, and plasma was transferred into 2 mL pre-labelled SARSTEDT cryotubes (*Aktiengesellschaft & Co, D*-*51588 Numbrecht, Germany*). A portion of this plasma was used to perform the single HIV and syphilis testing, as defined in the national algorithm. If testing was not done on the same day of specimen collection, plasma were kept at 2–8 °C for not more than 24 h before analysis. Test were carried out according to the manufacturer’s kit instructions that were embodied into respective standard operating procedures (SOPs). The presence of one band (C) indicated a negative result, while the (C) and HIV bands indicated a positive HIV result, the C and syphilis bands were indicative of positive syphilis test, and the C, HIV and syphilis bands indicated syphilis and HIV dual infection. Syphilis was confirmed using *Treponema pallidum* Hemagglutination assay (*BIOTEC Lab21 Healthcare Ltd, Dorset, UK*), while HIV was confirmed using the established algorithm in which samples were tested serially with; (a) Determine HIV-1/2 (*Abbott Laboratories, Abbott Park, Ireland*), (b) HIV 1/2 Stat-Pak™ (*Chembio Diagnostic Systems, Medford, New York 11763, USA*) and (c) Uni-Gold recombinant HIV-1/2 (*Trinity Biotech, Bray, Ireland*) assays. Using this approach, a negative first assay was considered negative, while a positive first assay was followed by testing using Statpak and confirmed with Uni-Gold Recombinant HIV1/2 assay (Trinity Biotech, Bray, Ireland). The test bands were interpreted appropriately by two independent laboratory technicians, were a discordancy was observed, a third read was considered. The flow of laboratory testing is summarized in Fig. 1.

Data were analyzed using frequency tables. The operational performance was determined based on measurement of Sensitivity, specificity, positive predictive value and negative predictive value of the Duo Kit were calculated. Sensitivity = True Positives (TP)/[TP + False Negatives (FN)]; Specificity = TN/[False Positives (FP) + TN]. Agreement between the test methods was assessed using the Cohen’s kappa coefficient (κ value). The feasibility of the SD Bioline HIV/Syphilis Duo assay was determined by ease of the test method, turnaround time and ease of result interpretation.

Ethical approval was obtained from the research and ethics committee of Clarke International University. Permission to carry on research was obtained from the District Health Officer, and Mayuge Health Centre III in-charge. This study was voluntary, and written informed consent was sought from each study participant.

### Results

The study enrolled 382 consented healthy pregnant women. Their mean age was 25.8 years (SD = 5.977) IQR = 9. 57. Majority (32.7%; N = 125) of the participants were aged 20–24 years, 52.1% (N = 199) had completed primary level, and 65.7% (N = 251) were in a monogamous relationship. Their socio-demographic characteristics are given in Table 1.

**Table 1:** Showing the socio-demographic factors of study participants (n = 382)

Variable	Frequency	Percentage
Age category (years)		
≤ 19	57	14.9
20–24	125	32.7
25–29	96	25.1
≥ 30	104	27.2
Religion		
Catholic	113	29.6
Protestant	107	28.0
Muslim	23	6.0
Pentecostal	123	32.2
Others	16	4.2
Education level		
None	61	16.0
Primary	199	52.1
Secondary	88	23.0
Tertiary	16	4.2
University	18	4.7
Self employed	241	63.1
Occupation		
Employed	49	12.8
Unemployed	92	24.1
Marital status		
Married	279	73.0
Divorced	26	6.8
Widowed	06	1.6
Others	71	18.6
Type of marriage		
Monogamous	251	65.7
Polygamous	69	18.1
Others	62	16.2
Exposure risk to HIV		
Yes	79	20.7
No	303	79.3
Exposure risk to syphilis		
Yes	143	37.4
No	239	62.6

Seven of the participants tested positive for HIV, giving a prevalence of 1.8% (95% confidence interval 1.23–2.41). Eight participants tested positive for syphilis, giving a prevalence of 2.1% (95% confidence interval 1.61–2.54). The prevalence of HIV and syphilis dual infection was 2 out of 382 (0.52%; 95% CI 0.37–0.92). Summary of the diagnostic performance is given in Table 2. Results of the Cohen’s Kappa are given in Additional file 1: Table S1.

**Table 2:** Showing the diagnostic performance of HIV–Syphilis SD Bioline Duo Kit

RDT	Infection	RDT result	Positive^a^	Negative^a^	Total
Diagnostic performance of HIV–Syphilis SD Bioline Duo Kit					
SD HIV/Syphilis Duo	HIV	Positive	7	0	7
		Negative	0	375	375
		Total	7	375	382
	Syphilis	Positive	8	0	8
		Negative	0	374	374
		Total	8	374	382

RDT	Infection	% Sensitivity (95% CI)	% Specificity (95% CI)		
Showing the operation performance of the HIV–Syphilis SD *Bioline* Duo Kit					
SD Bioline HIV–Syphilis Duo assay	HIV	100.0 (99.5 to 100.0)	100.0 (98.6 to 100.0)		
	Syphilis	100 (98.3 to 100.0)	100 (98.6 to 100)		

^a^The gold standard method for HIV was the Ministry of Health (Uganda) HIV testing Algorithm; while for Syphilis, it was the *Treponema pallidum* Hemagglutination Assay (TPHA)

### Discussion

There are numerous efforts to routinely screen for HIV and syphilis infections among pregnant women. This requires the use of highly sensitive, low cost and rapid point-of-care tests to aptly diagnose and initiate care. One such invention is the SD *Bioline* HIV–Syphilis Duo assay that comes with the desired attributes. From this study, the HIV prevalence of 1.8%, is less than the overall country prevalence of 6.2%, and also that among the Ugandan women of child bearing age that was reported at 7.5% [21]. The sizably lower prevalence of HIV than the national estimates is attributed in part to increased sexual health education initiatives [22]. The prevalence of syphilis was 2.1%, a value close to the nationally document prevalence of 2.2% among adult Ugandan [21]. In spite of the seemingly lower prevalence values of HIV and syphilis infections from this study, the rates remains unacceptably high, and may portend the global efforts towards the elimination of the dual infection. To this, there is need to upsurge detection of the dual infection and the associated sequelae [22–25].

The diagnostic performance of the SD *Bioline* HIV–Syphilis Duo Kit as measured by sensitivity and specificity was 100.0%. This is in agreement with previous studies that reported higher sensitivities for syphilis (100.0, and 99.7%, respectively) and comparable sensitivities for HIV (99.1, and 100%, respectively) [14, 26–30]. The observed high syphilis sensitivity of the SD Bioline Duo Kit is explained by the variance in the infection stage, as earlier studies suggested [31–33]. This study contributes to the growing evidence in support of high diagnostic performance of the assay in a resource-limited setting. This brings with sufficient evidence to implement the dual assay in abide to achieve the double elimination. The SD *Bioline* assay showed great attributes of use, including a shortened turnaround time (20 min), being user friendly, use of micro volumes that may warrant its performance using capillary blood sample. The assays still befits the economic pressure of limited resource setting as it costs about 2 United States Dollar versus 2 United States Dollar for TPHA and 3–10 United States Dollar for the standard HIV algorithm [14]. In addition, the assay could bridge the high workload of testing HIV and syphilis separately as seen in most health facilities, and this has proven ideal for our setting as personnel capacities remains below the required. Although the results of this study are reassuring of excellent operational performance of the assay, we did not evaluate its performance with capillary blood, in particular figure prick samples. Also, there were few number of positives for HIV and syphilis infections; this in a way makes the results of sensitivity and specificity to be biased. In addition, the study did not use the rapid plasma regain (RPR) to screen for active infections, and also gold standard polymerase chain reaction assays.

### Conclusion

This study reports an optimal operational performance of SD Bioline HIV/Syphilis Duo Kit, which reaffirms the already established scientific finding. This makes it suitable for the integration in the testing and treatment programs to ultimately eliminate the dual infection. With its price point and ease of use, it is irrefutable that this assay suites the operation in a limited resource setting to control and manage sexually transmitted infections. This RDT kit offers an opportunity for simultaneous screening of both HIV and syphilis in pregnant women across our antenatal care and prevention of mother to child transmission (PMTCT) programs. However, for country wide rollout, a performance evaluation of the national HIV test algorithm with a consideration of the SD Bioline HIV/Syphilis Duo RDT as a first test is recommended.

## Limitation of the study

There are short falls of this study, the use of venous blood which was centrifuged and tested in a laboratory as opposed to point-of care whole blood specimens obtained from a figure prick. Also, the study did not elucidate the PCR confirmation of the duo-infection as the gold standard diagnosis.

## Additional file

**Additional file 1: Table S1.** Results of the Cohen’s Kappa.

## Abbreviations

HIV: human immune deficiency virus
RDTs: rapid diagnostic tests
STIs: sexually transmitted infections
TPHA: *Treponema Pallidum* Haemagglutination Assay
WHO: World Health Organization
