# Supplementary material for: Laboratory assessment of SD Bioline HIV/Syphilis Duo Kit among pregnant women attending antenatal clinic Mayuge Health Center III, East central Uganda
Source: BMC Res Notes. 2019 Apr 25;12:238. doi: 10.1186/s13104-019-4272-6 (PMC6482528; doi:10.1186/s13104-019-4272-6)
Supplement: Supplementary file 1 — Additional file 1: Table S1. Results of the Cohen’s Kappa. [file 13104_2019_4272_MOESM1_ESM.docx]

**Table S1: Results of the Cohen’s Kappa**

| HIV/AIDS test kit | | | | |
| --- | --- | --- | --- | --- |
|  | Value | Asymp. Std. Error^a^ | Approx. T^b^ | Approx. Sig. |
| Measure of Agreement | -0.0187 | 0.00499 | -0.3648 | 0.715 |
| N of Valid Cases | 382 |  |  |  |
| *a. Not assuming the null hypothesis.* | | | | |
| *b. Using the asymptotic standard error assuming the null hypothesis.* | | | | |
| Syphilis test kit | | | | |
|  | Value | Asymp. Std. Error^a^ | Approx. T^b^ | Approx. Sig |
| Measure of Agreement | -0.0214 | 0.00535 | -0.4181 | 0.676 |
| N of Valid Cases | 382 |  |  |  |
| *a. Not assuming the null hypothesis.* | | | | |
| *b. Using the asymptotic standard error assuming the null hypothesis.* | | | | |
